# Recent Advances in Omics Technology in Food Flavor

**DOI:** 10.3390/foods15152637

**Published:** 2026-07-27

**Authors:** Yi Xiao, Xiulian Wang, Jia Zhang, Xinyu Hu, Wei Wang, Lili Ji, Ting Bai, Jiamin Zhang

**Affiliations:** 1 Meat Processing Key Laboratory of Sichuan Province, Sichuan Provincial Engineering Research Center of Meat Quality Improvement and Safety Control Technology, Chengdu University, Chengdu 610106, China; 2 College of Food and Biological Engineering, Chengdu University, Chengdu 610106, China

**Keywords:** multisystem integration, flavor control, mechanism analysis, causal framework, artificial intelligence

## Abstract

Flavor is a key determinant of food quality, and elucidating its formation mechanisms is of great significance for product optimization and satisfying consumer preferences. Traditional sensory analysis is inherently subjective, while instrumental analysis can objectively assess flavor components but provides limited insight into the formation mechanisms. This article adopts a narrative literature review to summarize the applications of omics technologies in food flavor research in recent years. It focuses on genomics, transcriptomics, proteomics, metabolomics, lipidomics, and multi-omics technologies. It points out that current studies remain largely confined to identifying differential molecular features, lacking in-depth exploration from molecular interactions to causal mechanisms. At the same time, single-omics methods can only provide partial information and cannot capture the multi-level regulatory networks of flavor formation. A causal inference framework for analyzing flavor mechanisms at the omics level through multi-omics integration and intervention experiments has been proposed. It emphasizes that artificial intelligence technology provides a promising solution for the interactive analysis of complex multi-omics datasets. Future research should focus on developing low-cost, high-throughput real-time analysis methods, cross-omics data fusion strategies, and the rigorous exploration of scientific causal relationships.

## 1. Introduction

Food flavor represents a comprehensive brain response to chemical and physical stimuli in food, mediated by taste, smell, and somatosensory sensations [1]. Its perception depends on multiple factors, including the detection of volatile flavor compounds (VOCs) by olfactory receptors [2], the response of taste buds to basic tastes (e.g., sour, sweet, bitter, spicy, salty, umami), and textural attributes such as chewiness [3,4]. Flavor formation arises from the combined action of numerous chemical components—for instance, citrus flavor is generated by a mixture of terpene-derived compounds [5]—and can be influenced by extraction or processing techniques [6].

As a key indicator of food quality and consumer acceptance, flavor research is of great significance in food science [7]. According to a recent market research report, the global food flavors and fragrances market was valued at approximately USD 335.8 billion in 2025 and is projected to exceed USD 575.2 billion by 2033, with a compound annual growth rate (CAGR) of 7.1% [8]. However, traditional approaches have primarily focused on individual molecules or single analytical levels, limiting a comprehensive understanding of the mechanisms underlying flavor formation [9,10], which results in a prolonged development cycle for new flavor products (typically ranging from 12 to 18 months) and substantial development costs (with the investment for a single product development reaching tens of thousands to millions of dollars) [11].

In this context, omics technologies (genomics, transcriptomics, proteomics, metabolomics, lipidomics) offer a high-throughput (HTP), systems-level alternative [12]. By simultaneously analyzing genomes, transcriptomes, proteomes, and metabolomes, these technologies enable a more comprehensive investigation of the biological pathways underlying flavor development [13]. Recent studies have demonstrated their potential: for example, Xuyue et al. [6] employed multi-omics (MO) to reveal how lipid oxidation during pickling contributes to salted egg yolk flavor, while Qian et al. [14] combined metabolomics with flavor profiling to investigate the effects of low-salt fermentation on sour meat quality and microbial community structure. These examples underscore the potential of omics to decipher flavor formation and its modulation by processing conditions. According to the search results from the ScienceDirect online database, the number of papers published based on the keywords “omics technology” and “food flavor” increased from 265 in 2021 to 1314 in 2025, and the number has reached 1044 by April 2026, demonstrating a rapid upward trend in both basic research and applied development.

Although several reviews have addressed omics in food science, for instance, Zhang Yue et al. reviewed the research progress of MO technologies in regulating the flavor of lactic acid bacteria (LAB) in fermented beverages and proposed a systematic framework for flavor regulation by LAB from the perspective of MO integration [15]. Sadaqat Ali et al. summarized recent advances in the application of lipidomics to chicken flavor research, and pointed out that significant research gaps remain between lipid group characteristics and sensory perception as well as consumer preferences [16]. Wei Ruan et al. summarized the metabolic dual characteristics of metabolomics in Chinese fermented foods, from aroma to off-flavors (OF), and emphasized that metabolomics is an indispensable analytical tool for systematically elucidating the biochemical pathways for the formation of ideal aromatic compounds [17]. These studies mainly focused on summarizing the omics analysis of individual or certain types of foods, mainly identifying the differences in metabolites or genes, but lacking in-depth verification of the causal relationships within the flavor biological regulatory network; the heterogeneity of data and the absence of standards for cross-omics integration have significantly hindered the improvement of the efficiency of mechanistic investigations. There is also a lack of strategies for selecting omics for different research goals (such as tracing, regulation, and prediction). At the same time, few have compared the roles of different omics layers (transcriptomics, proteomics, metabolomics, lipidomics) and their integration in flavor research.

In order to clarify the problems existing in the application of omics technologies in food flavor research, a narrative literature review method was adopted. This method enables a comprehensive investigation of research results on a specific topic and is more suitable for exploring innovative and recent phenomena [18]. Our aim is to provide an overview of the research on the formation or influence mechanisms of food flavors by various omics technologies, using an inductive approach. After identifying the topics, we repeatedly classified, grouped and differentiated them. The search covered research papers and reviews published from 2022 to 2026 in the ScienceDirect online database. The keyword “food flavor” was used in combination with the search terms “genomics”, “transcriptomics”, “proteomics”, “metabolomics”, “lipidomics”, and “multi-omics”. After manual screening, the final classification of the articles is as follows: genomics (15), transcriptomics (15), proteomics (12), metabolomics (23), lipidomics (11), multi-omics (15) (with overlaps). Based on the identified issues from the summary, we conducted a critical discussion by searching for relevant literature using these key words.

This article reviews the recent advances in the application of omics technologies to food flavor in terms of genomics, transcriptomics, proteomics, metabolomics, lipidomics and MO approaches. By summarizing the achievements and limitations of existing research techniques and evaluating the reliability and reproducibility of MO data, a multi-level analysis strategy integrating genomics, transcriptomics, metabolomics and proteomics is proposed. This strategy emphasizes the integration of MO data and the establishment of causal verification models, in order to analyze the dynamic network regulatory mechanisms of flavor formation, providing a reference framework for applying omics strategies to unravel flavor complexity and guide future research.

## 2. Development of Food Flavor Analysis Methods

Food flavor comprises both taste and aroma. Taste can reflect sweetness, sourness, saltiness, bitterness, umami, and spiciness. Aroma results from the response of olfactory receptors to VOCs, which mainly include alcohols, aldehydes, esters, ketones and sulfur-containing compounds [19,20]. These VOCs in food have a low detection threshold. Aroma-active compounds are a minority of the known volatile compounds. The odor activity value (OAV) is calculated by dividing the concentration of flavoring components in the food by their detection threshold. It is used to determine the aroma-imparting properties of flavor substances (the higher the OAV, the more significant its contribution). Generally, volatile compounds with an OAV > 1 are considered aroma-active compounds that contribute significantly to the overall aroma profile [21].

**Figure 1:**
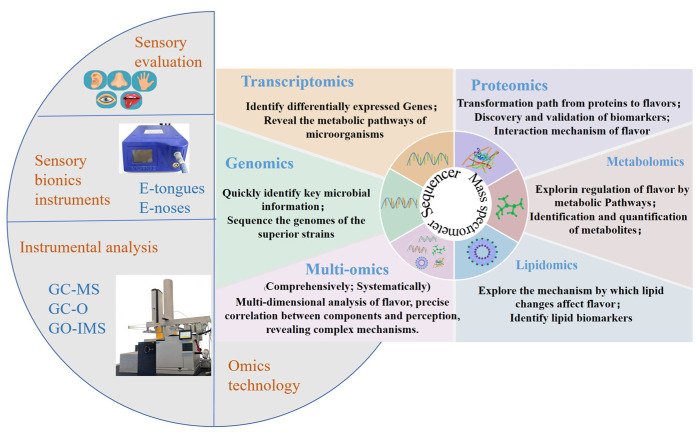
Development of food flavor analysis technology.

The development of food flavor analysis technologies has generally progressed through three stages (Figure 1). In the initial stages of flavor analysis, sensory evaluation was widely used to assess food attributes, including appearance, taste, and aroma. For example, Yiming et al. [22] selected evaluators to characterize rice flavor and analyze consumer acceptance. In addition to the selection of evaluators, professional sensory evaluation often requires several years of systematic training. For instance, Qiuyun et al. [23] employed trained sensory assessors to visually evaluate Jiangwei Yuandi liquor from the Chishui River Basin. Sensory evaluations are the most direct method for evaluating flavor, but they are affected by evaluators’ characteristics and may be highly subjective. More significantly, they cannot identify potentially harmful substances, making it difficult to conduct qualitative and quantitative analyses of flavor compounds.

In the second stage of flavor analysis, instrumental analysis methods that employ specialized instruments to characterize the chemical composition, content, chemical structure, and other information of a substance can achieve objective analysis of the data [24]. Novel sensory bionics instruments such as electronic tongues (E-tongues) and electronic noses (E-noses) utilize biomimetic sensor arrays to simulate animal olfactory and gustatory perception, enabling rapid identification of complex odor or taste profiles through pattern recognition. Their key benefits are rapid detection speed, simple operation, and elimination of complex pretreatment requirements, making them suitable for applications such as online monitoring, quality grading, and shelf-life assessment. For example, Stevan Jr et al. [25] used an E-nose to measure and analyze VOCs of four varieties of blueberry fermentation samples to distinguish different blueberry by-products, and Singh et al. [26] evaluated the shelf life of seven edible seeds by using an E-nose to discriminate VOC fingerprints of the seeds over 150 days of storage. However, these techniques cannot identify or quantify individual compounds and lack the capability to analyze structural information of specific components in mixed systems [27].

Gas chromatography–mass spectrometry (GC-MS) serves as the cornerstone technique for both qualitative and quantitative flavor analysis, combining the high resolution of chromatography with the structural identification capabilities of mass spectrometry. It enables precise quantification of VOCs and semi-VOCs while also facilitating the identification of key flavor components through spectral library searches and retention index correlation. For instance, Hailei et al. [28] identified 54 VOCs in thawed duck meat using headspace solid-phase microextraction (HS-SPME) and GC-MS, revealing that aldehydes are primarily responsible for the meat odor. However, GC-MS has limitations in detecting trace substances and fails to directly quantify each component’s actual sensory contribution to overall flavor. Gas chromatography olfaction assay (GC-O) integrates chromatographic separation with human olfactory assessment, allowing identification of odor-active compounds from complex mixtures. Through an olfactory port, trained assessors can record the odor characteristics and intensity of each chromatographic peak, establishing direct correlations between chemical signals and sensory perception [29]. While GC-O effectively identifies key aroma-active compounds and avoids unnecessary analysis of odorless substances, its results are susceptible to individual variability among assessors and lack quantitative precision. Gas Chromatography–Ion Mobility Spectrometry (GC-IMS) combines the high separation capabilities of gas chromatography with the rapid response of ion mobility spectroscopy (IMS), enabling the separation of volatile compounds based on their ion mobility characteristics. Compared to GC-MS, the most prominent features of GC-IMS are its high separation efficiency, fast detection speed, and convenient operation [29,30].

The combination of chromatography and mass spectrometry with sensory evaluation has led to the development of GC–olfactometry (GC-O) and GC-MS combined with OAV analysis. Guangyuan et al. [31] utilized an E-nose to characterize VOCs to differentiate perilla leaves under various pretreatment and drying conditions. When compared with GC-MS analysis, the results showed that the VOCs of perilla leaves could be better retained in environments with low drying temperatures and brief pretreatment times. Xiaoai [32] used GC-MS, an E-nose, and an E-tongue to analyze flavor characteristics and clarify the effect of noni fruit maturity on quality and flavor, indicating that noni fruits had distinctly diverse odors depending on maturation, with the largest alterations occurring between maturity levels 8 and 9; freshness, astringency, and sourness were the main flavor characteristics. Food flavor compounds can be more precisely identified and analyzed when E-tongues and E-noses are combined with GC-MS and other devices.

However, other techniques are required to fully understand the dynamic alterations and formation mechanisms of VOCs. Omics, including sequencing-based genomics and transcriptomics, as well as mass spectrometry-based proteomics, lipidomics and metabolomics, are HTP techniques used to measure various molecular components in biological samples [33,34]. In flavor research, metabolomics, proteomics, and lipidomics are frequently employed in investigating flavor component synthesis and regulatory mechanisms [35,36,37,38], whereas genomics and transcriptomics reveal differences in annotated genes affecting the metabolism of flavor compounds at the DNA and RNA levels, respectively [39,40]. These technologies can provide a more thorough grasp of the alterations and development of flavor components during food processing, storage, and consumption, offering theoretical support for enhancing food flavor and maintaining quality. Simultaneously, newly discovered food flavor substances can enrich flavor types and quality.

The examination of the evolution of food flavor analysis techniques reveals that sensory analysis, sensory biomimetic instruments, chromatographic instrument analysis, and omics technologies represent not only generational changes but also a gradual improvement in analytical capabilities and interpretation depth. Each of these technologies has unique and irreplaceable advantages while also exhibiting inherent limitations (Table 1). Sensory analysis, as the “gold standard”, provides overall perception, while chromatography–mass spectrometry and biomimetic instruments bridge the gap between sensory and quantitative analysis, enhancing objectivity. Omics technologies, on the other hand, provide insights into the molecular mechanisms underlying flavor formation at the genetic and other levels. These three methods are not simply substitutes but rather complementary and progressive: sensory analysis makes an initial judgment, instrument analysis locks in key substances, omics technology clarifies the regulatory mechanisms, and together they form a complete research loop from phenomenon to essence and back to verification. This evolution reflects the continuous improvement of analytical capabilities and interpretation depth and also indicates that the collaborative use of multiple technologies is the inevitable path to addressing the complexity of flavors.

## 3. Application of Omics Technology to Food Flavor

### 3.1. Application of Genomics (Metagenomics) to Food Flavor

Genomics consists of the aggregate description and measurement of every gene in an organism, the relationships between these genes, and their effects on the organism [41]. It is applicable for analyzing the genetic basis and flavor-related metabolic pathways of specific organisms (such as crops and fermentation strains), and its core methods include whole genome HTP sequencing, genome assembly and functional genomic analysis [42]. Metagenomics is the study of the combined genomes of all microorganisms in environmental samples [43]. It does not require isolation and cultivation and is applicable for studying the structure of complex microbial communities, the distribution of functional genes, and their collective contribution to flavor. In simple terms, genomics studies the complete genetic blueprint of a single species, while metagenomics studies all the genes of a group of species. However, whether involving a single strain or a complex microbial community, the biosynthesis and regulatory control of flavor metabolites ultimately operate within the hierarchical cascade: genes → transcripts → proteins → metabolites. This means that flavor formation is not the result of a single, specific chemical reaction; rather, it represents a broad metabolic output that cells or microbial communities have evolved to enhance environmental adaptation. Ying et al. [44] revealed the regulatory mechanism of MdVHP1-2 on the accumulation of flavor substances (organic acids, sugars), providing a key target for improving apple quality and developing new apple varieties. Ruijie et al. [45] used metagenomics to analyze the effect of probiotic inoculation on the flavor of low-salt dry-cured mackerel. Metagenomics identified the upregulation of genes encoding fatty acid (FA) metabolism enzymes, thereby explaining the generation of VOCs.

However, genomics alone cannot be used for the study of food flavor mechanisms; it needs to be integrated with other instruments or omics technologies to achieve the experimental goals. In food flavor research, metagenomics rapidly identifies key microbial groups, functional genes and metabolic pathways related to flavor synthesis in environmental samples (such as goji bud tea [46], high-temperature Daqu [47] and fermented broad bean paste [48]) through metagenomics, using genomic data to identify superior varieties (such as alcoholic [49], fermented sausages [50,51] and dry-cured hams [52]), and studying gene expression regulation to optimize food quality and flavor. Specifically, Zhao et al. [46] utilized metagenomics to rapidly screen for key microbial groups related to flavor reshaped by pile-fermentation (PF) in goji bud tea, selecting three microorganisms shaping flavor. Renau-Morata et al. [53] used genomics to analyze the genetic diversity of *Solanum* species and address the nitrogen limitation of cultivated tomatoes with sustainable production. They proposed candidate genes to increase biomass, fruit yield, and fruit quality traits under low nitrogen input in cultivated tomatoes. These studies have established a systematic flavor formation analysis strategy comprising two sequential phases: macroscopic screening to identify candidate targets, followed by microscopic analysis to confirm their functional roles. Specifically, the strategy integrates metagenomic analyses, including functional gene enrichment and microbial community network construction, to rapidly pinpoint key microbial taxa and metabolic pathways that exhibit positive correlations with flavor compound accumulation. Subsequently, genomic analyses of representative bacterial isolates are employed to precisely elucidate the molecular mechanisms underlying the biosynthesis and accumulation of flavor-related metabolites.

Overall, the combination of genomics and metagenomics enables a closed-loop study from the flavor potential of the entire microbial community to the regulation of individual genes for specific flavor substances. However, relying solely on genomics or metagenomics can only provide genetic potential and cannot reflect the actual metabolic changes. The MO integration combining the genome with the transcriptome, proteome and metabolome is increasingly being used to distinguish the differences between genes and expressed genes. Flavor is a dynamic equilibrium process; collecting samples at different time points to construct a dynamic tracking process of flavor formation, identifying the key regulatory points of flavor generation, provides systematic molecular tools for the rational design of food flavors, optimization of fermentation processes, and screening of high-quality genetic resources.

### 3.2. Application of Transcriptomics to Food Flavor

Transcriptomics is the comprehensive investigation of gene transcription and transcriptional regulation in living cells to study the synthesis and regulation mechanisms of VOCs at the RNA level. By analyzing the transcriptional differences among different varieties [54,55], microbial fermentation effects [56], and treatment conditions [57], the differentially expressed genes related to flavor synthesis and the upstream transcription factors are identified. This allows for a deeper understanding of synthesis and perception mechanisms, offering a theoretical foundation for improving food flavor. At the same time, it provides useful information for research on variety breeding, precise fermentation and process optimization [58]. The essence of transcriptomics lies in dynamically capturing the expression profiles of genes, which can be used to reveal the metabolic pathways of microorganisms. For instance, Jingya et al. [59] used transcriptomics to reveal Luzhou-flavor liquor’s primary lactic acid-producing microbes and metabolic processes during fermentation. The metatranscriptome can answer the question of what microorganisms are doing under specific conditions. It involves HTP sequencing and analysis of all the RNA transcripts of all microorganisms in the sample, and is used to identify the microorganisms that are related to the formation of key flavors during the natural fermentation of Suansun [60].

Transcriptome analysis is core to revealing the mechanism of flavor formation. The raw sequencing data must undergo strict quality control, including the removal of adapter sequences and low-quality reads, to ensure the reliability of subsequent analysis [61]. Subsequently, differential gene expression (DGE) was evaluated using the DESeq software v1.46.0. Usually, DGE is screened based on the threshold of |log_2_(fold change)| ≥ 1 and either the *p*-value or FDR (False Discovery Rate) ≤ 0.05 [62]. For the selected candidate genes, especially those related to flavor synthesis and the upstream transcription factors, the expression levels need to be verified through real-time fluorescent quantitative PCR (qRT-PCR). Additionally, functional classification can be performed using Gene Ontology (GO) classification and the Kyoto Encyclopedia of Genes and Genomes (KEGG) database [63]. Ultimately, through phenotypic experiments using gene knockout or overexpression mutant strains, the actual role of these genes in the synthesis of flavor substances is confirmed at the biological functional level, thereby converting computational predictions into verifiable mechanistic conclusions [43].

Although transcriptomics can provide rich gene expression information, flavor development involves multiple regulatory levels. It may be difficult to explain flavor formation solely based on transcriptomics data. Therefore, transcriptomics is usually combined with instrumental analysis or other omics technologies to analyze food flavor [64]. Kun et al. [65] explained flavor differences mediated by phenolic compounds in different loquat varieties through transcriptomics and biochemical analysis. Transcriptomics combined with genomics revealed key candidate genes affecting goat milk flavor [66]. Transcriptomics combined with targeted metabolomics revealed the expressed genes and metabolic pathways that affect the flavor of black tea processed with different withering methods [67]. Transcriptomics combined with proteomics reveals the mechanism of influence of microbial strains on the flavor of fermented sour rice [68].

Transcriptomics can reveal which biological pathways are activated, whereas metabolomics or proteomics can elucidate which compounds are actually accumulated and which proteins are actually functional. A single transcriptome is sufficient to generate hypotheses, but only when combined with biochemical, metabolic, protein or genomic data can the process of flavor formation be verified and mechanistically explained.

### 3.3. Application of Proteomics to Food Flavor

Proteomics investigates protein composition and dynamic changes in cells, tissues, or organisms at the proteome level. It can be applied to understand the key mechanisms underlying food flavors and to improve and optimize food quality and flavor [69,70]. Proteomics is mainly applied to investigate the influence of varieties, feeding methods, and processing treatments on flavor. At the methodological level, quantitative proteomics has evolved from traditional two-dimensional electrophoresis and isotope labeling techniques (e.g., Isobaric Tag for Relative and Absolute Quantitation (iTRAQ), Tandem Mass Tag (TMT)) [71] to high-throughput strategies such as label-free quantification (LFQ) and data-independent acquisition (DIA) [70]. Among these, DIA has garnered significant attention due to its excellent reproducibility and wide dynamic range; however, its performance remains highly dependent on the quality of the spectral library and subsequent algorithm optimization [72]. Nevertheless, the reliability of quantitative results is consistently constrained by reproducibility issues: batch variations in sample preparation, signal drift in chromatography–mass spectrometry systems, and the choice of normalization methods can all introduce systematic biases. To mitigate these issues, researchers typically employ hybrid internal standard calibration, randomized multi-batch experimental designs, and stringent quality control (QC) protocols to minimize the interference of technical variability on biological interpretations.

Proteins play a critical role in food flavor formation. On the one hand, as precursors or reactants, they directly participate in the formation of flavor through biochemical reactions. First, analyzing protein composition and abundance in food helps to understand the transformation pathways from proteins to flavor. Bowen et al. [73] employed proteomics to investigate how myofibrillar protein glycosylation is affected by protein hydrolysis, highlighting the connection between flavor production and glycosylation and protein hydrolysis. Proteomics can also be used for biomarker discovery and verification [74]. In-depth research and understanding of protein markers related to food flavor can provide better insight into nutritional value, safety, and its relationship with health [75]. Yao et al. [76] used liquid chromatography–tandem mass spectrometry (LC-MS) and label-free shotgun (LFS) proteomics to perform multivariate analysis of meat quality and the proteome of beef’s longus pectoralis and psoas muscles, finding 85 proteins related to tenderness, chewiness, viscosity, and flavor. All quality attributes were linked to the putative biomarkers PHKA1 and STBD1, illustrating the potential mechanisms regulating meat quality in beef. However, from discovery to validation, protein biomarkers still require targeted quantification in independent sample cohorts, such as using parallel reaction monitoring (PRM) to monitor the quantitative mercaptans in wine [77].

On the other hand, the interaction between proteins as the matrix components and VOCs affects the transmission and perception of flavor. At this time, proteins can serve as dynamic reservoirs and controlled-release carriers for volatile compounds, helping us understand the combination mechanism of flavor and optimize food quality. Di Chenna et al. [78] used proteomics to interpret how β-lactoglobulin binds to four representative goat milk flavor components. This finding helped clarify which flavor components interact with β-lactoglobulin, highlighting their binding specificity, and offered a theoretical foundation for preserving goat milk quality. Proteins are not merely quality indicators; they are more likely to represent the status of metabolic or structural pathways, thus enabling proteomics to shift from describing correlations to explaining mechanisms.

Current food flavor proteomics research still faces multiple analytical challenges: food matrices are highly complex; high-abundance proteins can mask and interfere with the detection of low-abundance regulatory proteins; there is no unified standard for handling missing data values, integrated multi-omics analysis lacks established workflows; and varying data collection and processing protocols across laboratories make it difficult to directly compare and generalize research findings. Overcoming these challenges requires establishing standardized reference materials, improving open data analysis platforms, and fostering interdisciplinary collaboration so as to more effectively promote the application of proteomics in the targeted improvement of food flavor and precise processing.

### 3.4. Application of Metabolomics to Food Flavor

Metabolomics can qualitatively and quantitatively analyze small-molecule metabolites in an organism in a targeted and non-targeted manner [79], identifying correlations between metabolites and physiological changes in the body and analyzing changes in food flavor. Targeted metabolomics involves the in-depth study of a specific metabolic pathway or metabolite to understand its changes in the body. In contrast, non-targeted metabolomics involves comprehensively detecting metabolites in a sample to comprehend the organism’s metabolic alterations [80]. Technologies such as lipidomics, bioomics, and flavoromics have evolved from metabolomics. For example, flavoromics is used to identify small molecules related to flavor based on metabolomics and conduct targeted research on metabolites related to flavor [81].

Metabolomics can identify and quantify volatile and non-volatile flavor-related metabolites, thereby assessing the quality of food and determining its freshness or maturity. For example, some AA metabolites may taste pleasant [82], while some FA metabolites may taste bitter [83], and the fresh smell gradually transforms into a foul odor during storage [84]. Metabolomics has also been used to statistically analyze the effects of varieties [85], age [86,87], origin [88], storage conditions [89], and processing techniques [89,90] on the metabolic profile, and to explore how the regulation of metabolic pathways affects the synthesis or degradation of flavor substances, thereby providing potential targets for flavor improvement [91]. For instance, Samuel et al. [92] investigated the mechanism of biosynthesis of the flavor of raw pecans, identified ten key metabolic pathways related to flavor formation, discovered that the lipoxygenase pathway might contribute to the formation of buttery, nutty and fruity flavors, that chlorogenic acid is a key precursor for the biosynthesis of bitter flavonoids, and that pyruvic acid is associated with the biosynthesis of 2,3-butanediol and 3-penten-2-one.

Therefore, metabolomics and its derivative technologies (especially flavoromics) provide systematic tools from molecular detection to pathway regulation for flavor science. They have direct application value in food quality monitoring (such as freshness, maturity, and authenticity identification) and the development of new flavor products. However, metabolomics studies often involve data containing thousands of characteristic peaks. Direct application of conventional *t*-tests (*p* < 0.05) or Variable Importance in Projection (VIP > 1) from partial least squares discriminant analysis without controlling the False Discovery Rate (FDR)—due to neglecting multiple hypothesis testing—yields highly unreliable results [93]. To mitigate this, the Benjamini–Hochberg method or more robust q-values can be employed to control FDR and reduce the proportion of false positives [94].

Meanwhile, at the experimental level, rigorous QC strategies are paramount for ensuring the reliability of metabolomic data [95]. These include incorporating mixed QC samples into injection sequences to monitor instrument response and retention time drift [96]; establishing blank solvents to eliminate systematic interference [97]; adding stable isotope internal standards to correct for sample preparation and matrix effects [98]; and employing unsupervised methods such as principal component analysis (PCA) to visually assess QC sample clustering patterns [99], thereby intuitively reflecting system stability. If significant batch effects are identified, post-correction algorithms like ComBat or Surrogate Variable Analysis (SVA) can be applied. Regarding metabolite structure annotation, the Metabolomics Standards Initiative (MSI) recommends a four-tier confidence hierarchy [100]: Level 1 (definitive identification), requiring a complete match with standard reference retention times and mass spectra; Level 2 (presumed structure), based on database or literature matches; Level 3 (presumed category), derived from chemical classification information; and Level 4 (unknown compound). Currently, most non-targeted flavor studies only achieve Level 2 or 3 annotations [100], necessitating explicit reporting of each differential metabolite’s annotation level and cautious interpretation of subsequent pathways to avoid overgeneralization.

### 3.5. Application of Lipidomics to Food Flavor

Lipidomics systematically analyzes all lipid species in cells, organs, and organisms. It focuses on their composition and distribution, functions, interactions with other biological molecules, and dynamic changes under physiological states [37,101,102]. Lipids are major precursors of volatile flavor compounds in food, and their content and composition significantly affect food flavor [103,104]. Lipidomics is widely used to explore the potential mechanisms underlying flavor and lipid content alterations, in addition to the characterization of food quality and functional properties, providing guidance for optimizing food processing technologies and storage conditions [105,106]. Xiongwei et al. [37] used lipidomics to unveil the mechanism underlying oxidative rancidity and volatile flavor alterations in watermelon seed kernels and found that the most significant metabolic routes were the GLP and glyceride pathways. Ji et al. [102] investigated the influence of salt on flavor development in lightly salted yellow croaker by combining multisensory evaluation and lipidomics, finding that key VOCs are produced when sodium chloride increases lipase activity, which facilitates the hydrolysis and oxidation of phospholipids. These findings indicate that lipids are not only the source of flavor substances, but also that their oxidation and hydrolysis reaction pathways during processing or storage are highly selective. Lipidomics can distinguish the contribution of each lipid category to flavor at the pathway level.

Lipidomics is helpful in identifying lipid molecules or VOCs that are highly correlated with specific flavor attributes or quality changes. For instance, styrene and 1-pentanol may be employed as prospective biomarkers to assess the rancidity of watermelon seeds [37]. In egg yolks with added DHA, the increase in certain phospholipids and aldehydes is directly related to the production of specific flavors [101]. These markers can not only be used for quality control, but also serve as regulatory targets for processing. Lipidomics is often combined with sensory evaluation and VOC analysis (such as GC-MS) [107,108,109], and this integrated approach helps to distinguish the flavor contributions from lipid sources from their interactions with other components (such as proteins and carbohydrates).

Overall, lipidomics has been widely applied to analyze lipid oxidation/hydrolysis pathways during food processing and storage, trace the origin of flavor precursors, and screen quality markers. It is also combined with sensory evaluation and GC-MS, enhancing the dimensions of flavor contribution analysis. However, its limitations are clear: most studies are correlation analyses without causal verification; the association between lipids and volatile compounds often ignores the interference of protein/carbohydrate interactions; and sensory validation mostly stays at the descriptive level, lacking dose–response and dynamic threshold assessment. Moreover, the influence of processing heterogeneity and matrix effects on lipid reaction pathways has not been systematically modeled. In the future, efforts should be made to strengthen multi-omics integration, dynamic kinetic modeling, and standardized sensory validation systems, promoting the transformation of lipidomics from correlation analysis toward mechanistic prediction and regulation.

### 3.6. Application of Multi-Omics to Food Flavor

MO refers to using HTP technologies to obtain data from different dimensions in biological samples and comprehensively measure and analyze the information. It includes genomics, transcriptomics, proteomics, metabolomics, and others, and aims to comprehensively and systematically understand the complexity and diversity of organisms. MO comprehensively studies the formation, change, and perception of food flavors from multiple perspectives, providing comprehensive datasets for food research and development, production, and safety assessment. Genomics reveals the genetic basis of food flavor synthesis [110], providing a blueprint for the flavor, but the genes themselves do not directly produce the flavor molecules. Transcriptomics investigates changes in transcription levels, further revealing the synthetic processes of VOCs [111] and reflecting the transcriptional activity of genes under specific temporal and spatial conditions, but the RNA level does not represent the final protein function. Proteomics studies protein expression and modification, providing further insights into VOC synthesis and regulation [73] by directly regulating the enzymatic activity that catalyzes the synthesis of VOCs. Metabolomics and lipidomics directly correspond to the perceived flavor substances. Through this longitudinal causal relationship, a causal chain from genetic potential to molecular phenotype was formed, answering the question of why a specific gene leads to the accumulation of specific flavor compounds under specific conditions. For example, Zhehui et al. [112] employed metabolomics and transcriptomics to investigate key genes and characteristic VOCs of a novel brown navel orange and found that octanal was the key VOC and CsVS was the key gene.

MO methods can also be used to investigate individual variations in food flavor perception. Different people may perceive a particular food’s flavor differently due to differences in gene and oral microbiome variations. The microbiome and genome can help us understand how food flavors are formed and forecast taste alterations in an organism, as genetic differences determine the threshold and subjective intensity of individuals for specific compounds, while the oral microbiome metabolizes precursor substances into different flavor molecules, altering the flavor experience during the eating process [113]. Metabolomics is used to analyze amino acids and peptides produced by the breakdown of dietary proteins [114], indicating that flavor is not an inherent property of the food but rather the result of the interaction between food molecules and the individual sensory system, shifting flavor research from average sensory evaluation to personalized flavor prediction based on MO characteristics.

Weifang et al. [115] evaluated the flavor of egg yolks from Taihe Silky and Hy-Line Brown chickens using metabolomics and lipidomics. The differences between the carcass group and the lipid group reflect the genetic and environmental influences on lipid metabolism, yolk deposition, and oxidative stability in the two types of chickens. The variation in VOCs explains the reason for consumers’ preference for local eggs. This cross-species comparative analysis demonstrates the lateral comparison capability of MO, which can be extended to all agricultural product origin traceability and quality evaluation, replacing traditional single-component detection.

MO technologies have been widely applied in the genetic basis analysis of food flavors, processing regulation, and individualized perception prediction, achieving a leap from “one-dimensional detection” to a “causal chain”. However, current research is still mainly based on correlation analysis, lacking functional validation of key regulatory nodes; the integration of MO data has not been standardized, and noise and batch effects interfere with the reliability of the conclusions. Sensory verification is generally insufficient, and most studies stop at compound identification, lacking direct coupling with dynamic sensory evaluation, which weakens the ecological validity of the results. In the future, longitudinal intervention experiments (such as gene editing) need to be strengthened, a multi-omics-sensory mapping model needs to be established, and the dynamic oral metabolic process needs to be incorporated to fill the explanatory gap between “molecular signals and real perception”, thereby promoting flavor science to move from a descriptive tool toward an operable precise design system.

## 4. Discussion

### 4.1. Comparative Analysis of Different Omics Technologies in Food Flavor Research

Table 2 summarizes the comparisons of various omics technologies in food flavor research, covering the analysis objectives, typical analysis platforms, analytical advantages and limitations, typical applications, and future prospects. By comparison, it is found that omics technologies provide an unprecedented molecular-level perspective for the mechanistic analysis of food flavors. Flavor formation involves multi-level processes including precursor compounds, enzymatic catalysis, heat-induced reactions (such as the Maillard reaction and lipid oxidation), and microbial metabolism [116,117,118,119]. Metabolomics identifies molecular components associated with flavor; association analyses with genomics or transcriptomics help pinpoint key genes [112]; proteomics enables the identification of enzymatic changes involved in flavor formation [73]; and lipidomics reveals direct alterations in flavor precursor compounds. A single omics technology is unable to systematically and completely describe the food flavor formation mechanism. It is necessary to employ MO methods to construct a gene-metabolite interaction network that links genetic potential to molecular phenotypes, thereby characterizing a unique flavor profile.

However, omics studies represented by metabolomics and transcriptomics often remain limited to pathway enrichment analyses—for instance, metabolic pathways linked to odor deterioration primarily include linoleic acid metabolism, as well as alanine, aspartate, glutamate metabolism, and nucleotide metabolism [84]. Such analyses primarily provide statistical associations rather than mechanistic explanations of specific biochemical processes. Meanwhile, omics technologies are still restricted to particular food matrices or processing techniques, as high equipment investment and technical barriers, strict experimental design and quality control, complex data analysis, and relatively high research costs limit their widespread application in a complete closed-loop chain. Yizhen et al. [120] investigated how the flavor of various mackerel varieties changed after freezing using MO, uncovering metabolic pathways and biomarkers linked to the emergence of fishy odor but lacking a comprehensive evaluation of target metabolite abundances and other quality parameters such as safety. De Flaviis et al. [121] noted that omics is being increasingly employed to study the impact of ingredients, style, yeast, and wheat variety on the flavor of wheat craft beer; however, more advanced computational methods are required for data integration and interpretation in the future.

**Table 2:** Comparisons of various omics technologies in food flavor research.

Techniques	Targets	Representative Analytical Platforms	Advantages	Limitations	Typical Applications	Future Perspectives	Reference
Genomic	The entire DNA sequence of an organism reveals the genetic potential and regulatory genes for flavor formation	High-throughput sequencing platforms (such as Illumina).	Reveals flavor-related genes; predicts metabolic pathways and regulatory networks	Only provides “possibilities” and cannot directly reflect the actual changes in flavor substances; the data analysis is complex	Analysis of gene expression related to flavor synthesis [122]; Search for the core microorganisms related to the fermentation flavor [52,123]	Provide targets for precise flavor breeding and gene editing; integrate with metabolomics and transcriptomics to construct a “gene–protein–metabolite” regulatory network.	[42,43,44,45,46]
Transcriptomics	The expression products (RNA) of all genes in a specific time and space reflecting the active transcription status of the genes	RNA sequencing (RNA-seq)	Dynamic monitoring of changes in gene expression during flavor formation; identification of key regulatory pathways and transcription factors	Transcriptional level changes are not necessarily directly related to protein expression or metabolite content	Identification of differentially expressed genes related to flavor synthesis and upstream transcription factors [63,124]; Dynamic capture of gene expression profiles to reveal the metabolic pathways of microorganisms [125]	Serve as the bridge connecting “genotype” and “phenotype”; integrating machine learning to predict changes in flavor substances	[54,55,56,57,58,59,60,64,65,66]
Proteomics	All proteins in cells or tissues, including their expression levels, modifications and interactions	LC-MS/MS, 4D-DIA quantitative proteomics	Directly analyzes the molecules that govern executive functions (enzymes and structural proteins); reveals the direct contribution of protein degradation to flavor (such as the production of umami peptides).	The proteome is highly dynamic with a wide range of abundances; detecting low-abundance regulatory proteins remains a challenge; the analysis costs are relatively high	Study the relationship between protein degradation and flavor formation [82,83]; reveal the impact of protein–flavor compound interactions on the transmission and perception of flavor [78]	Combined with peptidomics to systematically analyze the generation pathways of flavor-active peptides	[69,70,71,72]
Metabolomics	All the small molecule metabolites (volatile and non-volatile) within the biological system constituting the ultimate material basis for flavor formation	GC-MS, LC-MS, Nuclear Magnetic Resonance (NMR)	Directly reflects changes in flavor substances; at the same time, it detects a large number of differential metabolites and analyzes the metabolic pathways.	The metabolite types are numerous and their physicochemical properties vary greatly, making it difficult to cover all aspects with a single method; the challenges in data processing and annotation are significant	Identification of key flavor compounds in food (such as amino acids, organic acids, aldehydes, ketones) [100]; analysis of dynamic change patterns of flavor substances during the fermentation and processing processes [101]	Build a powerful flavor prediction model through machine learning; deeply associate with sensory omics to quantify the “flavor–perception” relationship	[79,80,81,95]
Lipidomics	All lipid molecules in the organism, the contribution of lipid oxidation and degradation to flavor	UPLC-MS/MS	Precisely analyzes the flavor derived from lipids; identifies the key lipid precursors	The lipid metabolism network is complex and greatly influenced by the food matrix; data analysis and integration are also very challenging	Study the effects of lipid hydrolysis and oxidation under different processing or storage conditions on flavor [126]; identification of lipid molecules that are highly correlated with specific flavor characteristics or quality changes [127,128]	Combined with metabolomics, it clearly depicts the complete metabolic pathway from lipid precursors to volatile flavor substances; guide process optimization and flavor regulation in the development of precise nutritional foods	[37,101,102,103,104,105,106,107,108,109]
Multimodal integration	Systematically correlates information at multiple levels (genes–proteins–metabolites), and comprehensively analyzes the mechanism of flavor formation	Multimodal data integration platform + Bioinformatics analysis + Machine learning	Overcomes limitations of a single omics approach and achieves a closed-loop analysis from “cause” to “effect”; discovers more reliable biomarkers	The data volume is extremely large and the data types are heterogeneous; the integration analysis algorithms and standardization processes are not yet mature; the implementation cost is high	Screen and identify the core marker combinations for different varieties, regions and processed products [129,130,131]; investigation of individual differences in food flavor perception [106]; analysis of the relationship between key microorganisms in fermented products and flavor formation [39,132,133]	From genetic potential to molecular phenotype, reveal the gene–metabolite interaction network that determines unique flavors; represent the core trend of food flavor research; drive the food industry to achieve digital and intelligent flavor design and precise quality control	[110,111,112,113,121]

### 4.2. The Limitations and Verification Requirements of Omics-Based Flavor Association Analysis

Omics technologies are fundamentally association-based technologies, and statistical correlation does not equate to causal conclusions in biochemistry. Directly attributing differentially abundant metabolites identified in flavor studies to specific sensory attributes lacks a scientific basis. Without knockout experiments at the genetic or transcriptomic level, enzymatic inhibition assays in proteomics, or omics studies validated through sensory reconstitution or biosensor validation, such conclusions can only be regarded as hypotheses rather than facts. For instance, directly labeling aldehydes—whose abundance positively correlates with lipid oxidation during heating and the production of volatile compounds—as key aroma components overlooks the possibility that these compounds may also serve as common markers for other authentic aromatic substances [127].

**Figure 2:**
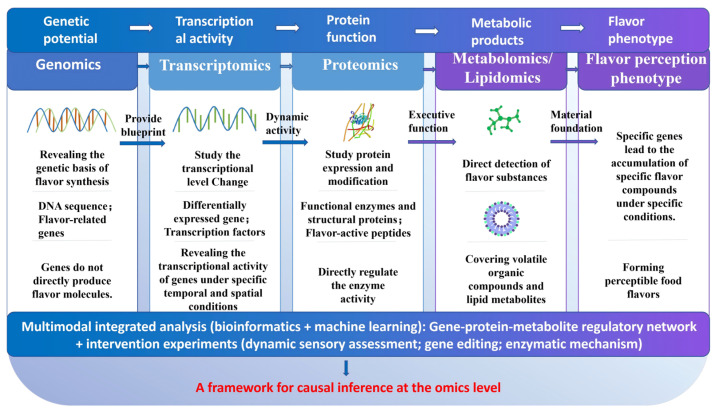
A framework for causal inference at the omics level.

Addressing these challenges requires intervention experiments and the establishment of an omics-based causal inference framework (Figure 2). Many omics-based flavor research papers rely solely on mass spectrometry identification and database annotation to claim the “discovery of flavor biomarkers” [36]; such findings, lacking sensory reconstitution validation, essentially remain chemical difference analyses, rendering their flavor relevance conclusions highly questionable. Flavor perception involves not only compound concentrations but also matrix effects, agonistic or antagonistic interactions with taste receptors, cross-modal integration, and central processing. For example, lipidomics studies have demonstrated a significant increase in PC during heating [107], yet whether this correlates directly with the sensory perception of oiliness requires further validation through sensory testing and receptor function experiments. Omics technologies are being employed to elucidate the molecular interactions between flavor compounds and receptors in the oral cavity, combined with electroencephalography (EEG) techniques to identify and evaluate neurophysiological aspects of taste perception in consumers [134]. These approaches can also measure kinetic parameters of key enzymes (e.g., Km, Vmax), conduct metabolic flux control analyses to quantify each reaction’s contribution to final products, and determine the subcellular localization of enzymes and metabolites for a comprehensive understanding of enzymatic mechanisms [135]. The CRISPR/Cas9 genome editing technology inactivated the CAR1 gene in brewing yeast strains. The results showed that both complete deletion and a premature termination codon insertion significantly increased the production of isopentanol (with tropical fruit aroma) and phenylethanol (with floral aroma) [136].

### 4.3. Artificial Intelligence Empowers Flavor Omics Data Analysis

The core value of MO integration lies in revealing the interactions among different molecular levels, and artificial intelligence (AI) provides a powerful tool to achieve this goal. AI has shown promising applications in food preservation, protein engineering, etc [137,138]. In food flavor research, traditional algorithms such as principal component analysis (PCA), partial least squares (PLS), random forest (RF), and support vector machine (SVM) are also used. These methods have low data requirements and fast training speeds, although they face the limitation of restricted predictive performance [81]. With the emergence of ensemble learning (eXtreme Gradient Boosting (XGBoost) and Light Gradient Boosting Machine (LightGBM)) and deep learning methods (Graph Convolutional Networks (GCN), Artificial Neural Network (ANN)), the SensoryGAN framework combines GCN with genetic algorithm (GA) to conduct molecular embedding learning on 47 essential VOCs, achieving a prediction accuracy of 86.14%, and conducting molecular reverse design under the constraint of target sensory attributes [139]. This “demand-driven” design paradigm provides a new strategy for traditional flavor development relying on empirical screening. In soy sauce flavor research, the eXtreme Gradient Boosting–Shapley Additive exPlanations (XGBoost-SHAP) model not only achieves a classification accuracy of >0.90, but also clearly reveals the chemical contributors to each sensory attribute, providing clear molecular targets for targeted flavor regulation [140].

Specifically, PCA and PLS are suitable for dimensionality reduction and linear relationship analysis, but they are sensitive to nonlinear relationships and prone to interference by outliers; RF and SVM perform robustly with small samples, but RF is prone to overfitting on high-noise data, while the selection of SVM kernel functions is subjective and has limited generalizability; XGBoost and LightGBM are good at handling sparse high-dimensional data, but parameter tuning is complex, and improper use may still cause overfitting, and they lack external validation across datasets; GCN can capture molecular graph structure features, but the model complexity is high, it is sensitive to molecular conformation changes, and its prediction results often cannot correspond to actual taste thresholds and dynamic perception differences.

Similarly, interpretable artificial intelligence (XAI) methods—such as SHAP (SHapley Additive Explanations) analysis—have been applied in flavor studies, including the prediction of sensory preferences for fermented pomegranate juice [141], the classification of baijiu quality grades [142], and coffee quality assessment [143]. By quantifying the contribution of each feature to prediction outcomes, these methods transform models from “black boxes” into visual tools. Consequently, AI-based machine learning techniques offer highly promising breakthrough solutions for the identification and processing of multi-omics data.

However, the application of AI-integrated MO technologies in food flavor research remains in its early exploratory phase. Unlike drug discovery and systems biology, where standardized data workflows and model validation frameworks have already been established [144], flavor studies lack unified methods for characterizing dynamic processes and quantitative perception systems, resulting in significant discrepancies between model predictions and actual consumer experiences. It is essential to guard against “overreliance on technological capabilities” and avoid equating molecular-level analyses with complete control over flavor quality.

The true value of omics technologies lies not in generating lengthy lists of differences but in their ability to explain why a given process makes food taste fresher, more aromatic, and richer. The field urgently needs to shift from descriptive analysis to mechanistic elucidation, elevating omics hypotheses into verifiable causal models. Only by incorporating sensory validation, receptor function testing, statistical robustness, and multi-level biological interpretation into experimental design can omics evolve from a tool for “discovering differences” into an engineering platform for “rational flavor design” This transformation requires not only advancements in technical methodologies but also researchers’ consistent commitment to rigorously investigating causal relationships throughout problem formulation, experimental verification, and result interpretation.

## 5. Conclusions

Omics technologies offer systematic, integrative approaches to food flavor research, enabling the comprehensive characterization of flavor compounds, microbial community dynamics, gene functions, and metabolic pathways. These technologies facilitate mechanistic insights into flavor formation, support quality optimization strategies, and enhance the capacity to evaluate food quality, freshness, and safety. Their high-throughput capabilities and automation features enable efficient large-scale sample processing, minimize analytical errors, and significantly improve experimental efficiency and reproducibility. Nevertheless, several challenges remain, including high operational costs, technical complexity, limited analytical sensitivity, bottlenecks in data integration and interpretation, and insufficient causal validation. In particular, data interpretation based on artificial intelligence must be strictly complemented by biological or chemical experimental validation to prevent overinterpretation and ensure scientific robustness.

Future research efforts should prioritize the establishment of standardized, multi-omics workflows; harmonization of preprocessing protocols; adherence to internationally recognized reporting standards; and the public deposition of raw data to strengthen reproducibility and enable robust meta-analyses. The integrated research strategies formed by combining hypothesis-driven experimentation with iterative experimental validation are essential to transform observed correlations into experimentally substantiated causal mechanisms. From a technological standpoint, portable omics platforms and real-time monitoring systems hold promise for on-site screening of flavor biomarkers and dynamic, process-responsive regulation of food manufacturing. However, broader industrial adoption remains constrained by economic factors (e.g., capital investment and operational costs), workforce training requirements, difficulties in system integration, and evolving regulatory frameworks, necessitating coordinated, cross-sectoral collaboration and rigorous cost–benefit analyses.

Collectively, omics technologies are transforming food flavor research from static compositional profiling toward dynamic, mechanism-informed process control. Through standardized analytical pipelines, AI-assisted multi-omics data integration, miniaturized sensing platforms, and real-time monitoring capabilities, this field is poised to deliver predictive models for flavor development, objective metrics for freshness assessment, and science-based frameworks for end-to-end quality assurance—ultimately providing scalable, practical solutions that align with both industry imperatives and evolving consumer expectations.

## Figures and Tables

**Table 1:** Comparison of food flavor analysis methods.

Content	Sensory Analysis [23]	Sensory Bionics Instruments [26]	Chromatographic Instrumental Analysis [28]	Omics Technologies [35,36,37,38]
Capability	Overall perception and judgment	Rapid and non-destructive overall discrimination	Qualitative and quantitative chemical components	Elucidation of mechanism pathways
Objective	Problem anchoring/issue identification	Quality rapid detection	Substance confirmation	Mechanism exploration
Limitations	Highly subjective,	Inability to conduct quantitative analysis	Lack of mechanism explanation	Absence of causal verification, risk of false positive
Requirements	Training and standardization	Reliance on control models	Standards and calibration curves	Intervention experiments + sensory reconstruction + FDR correction

## Data Availability

No new data were created or analyzed in this study. Data sharing is not applicable to this article.
